# Quantitative culture of endotracheal aspirate and BAL fluid samples in the management of patients with ventilator-associated pneumonia: a randomized clinical trial* **

**DOI:** 10.1590/S1806-37132014000600008

**Published:** 2014

**Authors:** Ricardo de Amorim Corrêa, Carlos Michel Luna, José Carlos Fernandez Versiani dos Anjos, Eurípedes Alvarenga Barbosa, Cláudia Juliana de Rezende, Adriano Pereira Rezende, Fernando Henrique Pereira, Manoel Otávio da Costa Rocha

**Affiliations:** Federal University of Minas Gerais, School of Medicine, Department of Pulmonology and Thoracic Surgery, Belo Horizonte, Brazil. Department of Pulmonology and Thoracic Surgery, Federal University of Minas Gerais School of Medicine, Belo Horizonte, Brazil; University of Buenos Aires, Hospital de Clínicas, Buenos Aires, Argentina. Hospital de Clínicas, University of Buenos Aires, Buenos Aires, Argentina; Hospital Madre Teresa, Belo Horizonte, Brazil. Intensive Care Unit, Hospital Madre Teresa, Belo Horizonte, Brazil; Hospital Madre Teresa, Belo Horizonte, Brazil. Laboratory of Microbiology, Hospital Madre Teresa, Belo Horizonte, Brazil; Hospital Madre Teresa, Department of Radiology, Belo Horizonte, Brazil. Department of Radiology, Hospital Madre Teresa, Belo Horizonte, Brazil; Hospital Madre Teresa, Department of Pulmonology and Thoracic Surgery, Belo Horizonte, Brazil. Department of Pulmonology and Thoracic Surgery, Hospital Madre Teresa, Belo Horizonte, Brazil; Federal University of Minas Gerais, School of Medicine, Postgraduate Center, Belo Horizonte, Brazil. Postgraduate Center, Federal University of Minas Gerais School of Medicine, Belo Horizonte, Brazil; Federal University of Minas Gerais, School of Medicine, Belo Horizonte, Brazil. Postgraduate Program in Health Sciences, Infectology and Tropical Medicine, Federal University of Minas Gerais School of Medicine, Belo Horizonte, Brazil

**Keywords:** Bronchoalveolar lavage fluid/diagnosis, Respiratory aspiration, Pneumonia, ventilator-associated

## Abstract

**OBJECTIVE:**

To compare 28-day mortality rates and clinical outcomes in ICU patients with ventilator-associated pneumonia according to the diagnostic strategy used.

**METHODS:**

This was a prospective randomized clinical trial. Of the 73 patients included in the study, 36 and 37 were randomized to undergo BAL or endotracheal aspiration (EA), respectively. Antibiotic therapy was based on guidelines and was adjusted according to the results of quantitative cultures.

**RESULTS:**

The 28-day mortality rate was similar in the BAL and EA groups (25.0% and 37.8%, respectively; p = 0.353). There were no differences between the groups regarding the duration of mechanical ventilation, antibiotic therapy, secondary complications, VAP recurrence, or length of ICU and hospital stay. Initial antibiotic therapy was deemed appropriate in 28 (77.8%) and 30 (83.3%) of the patients in the BAL and EA groups, respectively (p = 0.551). The 28-day mortality rate was not associated with the appropriateness of initial therapy in the BAL and EA groups (appropriate therapy: 35.7% vs. 43.3%; p = 0.553; and inappropriate therapy: 62.5% vs. 50.0%; p = 1.000). Previous use of antibiotics did not affect the culture yield in the EA or BAL group (p = 0.130 and p = 0.484, respectively).

**CONCLUSIONS:**

In the context of this study, the management of VAP patients, based on the results of quantitative endotracheal aspirate cultures, led to similar clinical outcomes to those obtained with the results of quantitative BAL fluid cultures.

## Abstract (pt)

**OBJETIVO:**

Comparar a mortalidade em 28 dias e desfechos clínicos em pacientes com pneumonia associada à ventilação mecânica (PAVM) internados em UTI conforme a estratégia diagnóstica utilizada.

**MÉTODOS:**

Ensaio clínico randomizado prospectivo. Dos 73 pacientes incluídos no estudo, 36 e 37, respectivamente, foram randomizados para a realização de LBA ou aspiração traqueal (AT). A antibioticoterapia inicial baseou-se em diretrizes e foi ajustada de acordo com os resultados das culturas quantitativas.

**RESULTADOS:**

A taxa de mortalidade em 28 dias foi semelhante nos grupos LBA e AT (25,0% e 37,8%, respectivamente; p = 0,353). Não houve diferenças entre os grupos em relação a duração da ventilação mecânica, antibioticoterapia, complicações secundárias, recidiva de PAVM ou tempo de permanência hospitalar e na UTI. A antibioticoterapia inicial foi considerada adequada em 28 (77,8%) e 30 (83,3%) dos pacientes nos grupos LBA e AT, respectivamente (p = 0,551). A mortalidade em 28 dias não se associou com a adequação da antibioticoterapia inicial nos grupos LBA e AT (tratamento apropriado: 35,7% vs. 43,3%; p = 0,553; e tratamento inapropriado: 62,5% vs. 50,0%; p = 1,000). O uso prévio de antibióticos não interferiu no rendimento das culturas nos grupos AT e LBA (p = 0,130 e p = 0,484, respectivamente).

**CONCLUSÕES:**

No contexto deste estudo, o manejo dos pacientes com PAVM, baseado nos resultados da cultura quantitativa do aspirado traqueal, resultou em desfechos clínicos semelhantes aos obtidos com os resultados da cultura quantitativa do LBA. (Registro Brasileiro de Ensaios Clínicos - ReBEC; número de identificação RBR-86DCDX [http://www.ensaiosclinicos.gov.br])

## Introduction

Ventilator-associated pneumonia (VAP) is a common complication of mechanical ventilation (MV), with a significant mortality rate, especially when associated with potentially antibiotic-resistant microorganisms.^(^ 1 ^-^ 3 ^)^ Early appropriate antibiotic therapy is associated with better outcomes, including a reduction in mortality.^(^ 4 ^-^ 6 ^)^

The microbiological diagnosis of VAP can be reached by invasive methods, such as fiberoptic bronchoscopic protected specimen brush (PSB) and BAL, or by noninvasive methods, such as endotracheal aspiration (EA). The former methods demand expert personnel, have potential complications, and are not promptly available; the latter methods can be readily performed, being also cost-effective and less invasive. Ideally, both specimens can be quantitatively cultured, aiming at reducing inappropriate treatment and the selection of multiresistant organisms.^(^ 7 ^,^ 8 ^)^ Few randomized trials examining the impact of microbiological information on clinical outcomes related to the diagnostic strategies have shown no substantial differences in mortality and etiologic diagnosis, although differences in the therapeutic protocols prevent direct comparisons among them.^(^ 9 ^-^ 11 ^)^

The objective of the present study was to compare the 28-day mortality rates in patients with microbiologically confirmed VAP. The patients were randomly assigned to two groups: LBA group, in which diagnostic work-up was carried out using BAL; and EA group, in which diagnostic work-up was carried out using EA. All of the samples of both groups were quantitatively cultured. Secondary outcomes included the length of ICU stay, adverse events due to sampling techniques, the appropriateness of antibiotic therapy and its impact on mortality, modifications in the antibiotic therapy, occurrence of secondary sepsis, occurrence of severe sepsis, occurrence of septic shock, VAP recurrence, and the need for additional diagnostic procedures.

## Methods

### Study population

The study population comprised consecutive adult patients (≥ 18 years of age) with a microbiologically confirmed VAP episode who had been admitted to the ICU of the institution.

The patients under MV for at least 48 h who presented with new or progressive pulmonary infiltrates on chest X-rays plus at least two of the following criteria-fever ≥ 38°C, purulent tracheal secretions, and leukocytosis ≥ 10,000 cells/mm^3^ or leukopenia ≤ 4,000 cells/mm^3^-were screened for enrollment. These patients were randomized to undergo BAL (BAL group) or EA (EA group). Both BAL fluid (BALF) and endotracheal aspirate samples were cultured. The patients whose cultures were positive-i.e., ≥ 10^4^ CFU/mL in BALF cultures and ≥ 10^5^ CFU/mL in quantitatively endotracheal aspirate cultures (QEACs)-were included in the study.

Patients with previously suspected nosocomial respiratory infection were enrolled only if that infection had been considered clinically and radiographically resolved. The exclusion criteria were a diagnosis of AIDS and inappropriate respiratory samples (> 10 squamous cells in the lower field magnification in endotracheal aspirate smears or > 1% bronchial cells in BALF smears). The protocol was approved by the research ethics committees of the institutions. Informed written consent was obtained from a close relative or a legal guardian of all subjects. Each patient participated only once in the study.

### Study design, setting, and procedures

Between August of 2000 and January of 2003, this randomized clinical trial was conducted in a 20-bed medical and surgical ICU at a 200-bed tertiary hospital (the *Hospital Madre Teresa*, located in the city of Belo Horizonte, Brazil) under the auspices of the Postgraduate Program in Infectious Diseases and Tropical Medicine of the Federal University of Minas Gerais, also located in the city of Belo Horizonte.

The hypothesis was that the mortality rates and the secondary outcomes would be similar between the two study groups, regardless of the sampling method adopted.

The randomization was performed independently by one ICU administrative staff member.

Age, gender, reason for ICU admission, prior use of antibiotics, and clinical laboratory data, as well as a lung injury score, a radiological score, and the Acute Physiology and Chronic Health Evaluation II score on the day of inclusion, were also recorded. Two blood samples were drawn for bacterial cultures immediately before the retrieval of respiratory secretion specimens. The respiratory samples were quantitatively cultured for aerobic microorganisms, and diagnostic thresholds of ≥ 10^4^ CFU/mL and ≥ 10^5^ CFU/mL being adopted for BALF cultures and QEAC, respectively. Respiratory viruses, *Legionella pneumophila*, *Chlamydia *spp. and *Mycobacterium pneumoniae *were not investigated. Strains of *Pseudomonas aeruginosa*, *Enterobacter *sp., *Acinetobacter *sp.*, Stenotrophomonas maltophilia *and methicillin-resistant* Staphylococcus aureus* (MRSA) were considered potentially resistant microorganisms. *Candida *sp. were considered to be colonizing microorganisms.

### EA and BAL procedures

An ICU respiratory therapist performed the EA procedure. A detachable 40-cm catheter was inserted through the endotracheal tube through a swivel adaptor without suctioning until it had reached at least 30 cm of insertion. The specimen was drawn with a sterile collector (Specimen Trap, St. Louis, MO, USA) and immediately sent to the laboratory for cytological and microbiological analyses.^(^ 8 ^)^

One of the authors of the present study performed all bronchoscopies and BALF collection procedures. After sedation, curarization, and adjustment of ventilatory settings, BALF was collected using a fiberoptic bronchoscope without the instillation of anesthetics or suctioning of secretions, as described in one study.^(^ 8 ^)^ The bronchoscope (Olympus BF1T20D; Olympus Optical, Tokyo, Japan) was inserted through the endotracheal tube and wedged in a subsegmental bronchus. Five 20-mL sterile saline aliquots at room temperature were infused and manually aspirated with a 20-mL volume syringe.^(^ 12 ^)^ The first aliquot was discarded, and the others were pooled and immediately sent to the laboratory. Pulse oximetry, electrocardiogram, and ventilatory parameters were monitored throughout the procedure.

For quantitative cultures, endotracheal aspirate samples were managed using a calibrated loop. Cytometry, cytology, and smear microscopy (May-Grünwald-Giemsa and Gram stainings) were performed, and the proportion of cells containing intracellular organisms (ICOs) was calculated after the counting of 300 cells at ×1000 magnification. Endotracheal aspirate samples were considered valid for culture if < 10 squamous epithelial cells and > 25 neutrophils were present. The adopted QEAC cut-off point for positivity was 10^5^ CFU/mL. BALF samples were considered appropriate for culture when < 1% of bronchial cells were found. After cytocentrifugation, the proportion of cells containing ICOs was calculated (May-Grünwald-Giemsa stain). BALF cultures that yielded ≥ 10^4^ CFU/mL were considered positive. A therapeutic protocol was employed in all of the cases, in accordance with the American Thoracic Society guidelines (adapted to the local epidemiology and microbial prevalence).^(^ 1 ^)^

The duration and adjustments of antibiotic therapy were based on the results of quantitative culture (BALF or endotracheal aspirate depending on the group), the clinical course, and the severity of the disease, at the discretion of the attending physician. Nonresponding patients were re-evaluated using the same initial strategy in order to establish a secondary diagnosis. Inappropriate initial antibiotic therapy was defined as the presence of bacteria resistant to or not covered by the initial antibiotic regimen. Radiological analyses were carried out independently by one of authors of the present study in accordance with a previously described methodology.^(13)^

### Statistical analysis

Continuous data were reported as mean ± SD or median and interquartile range. Categorical data were compared using the chi-square test or Fisher's exact test. The Student's t-test or the Mann-Whitney test was used for the comparison of continuous variables. Sample size was estimated at 76 patients in order to detect a difference in the 28-day mortality rate of at least 30 percentage points between the groups studied, with a power of 0.80 and a level of significance of p < 0.05.

## Results

Of the 80 screened patients, 73 were included in the study. The reasons for the exclusion of the 7 patients were as follows: negative cultures, in 2 patients; declining to participate in the study, in 2; and invalid respiratory samples, in 3 (2 endotracheal aspirate samples and 1 BALF sample). Thus, 73 patients were enrolled and randomized into the EA (n = 37) and the BAL groups (n = 36). Of the 73 patients, 47 (64.8%) were female. In the EA and BAL groups, respectively, the mean ages were 67.12 years and 64.49 years (p > 0.05; Table 1). The two groups were also similar regarding demographic data, prognostic scores, clinical data, and laboratory data. Of the 73 patients, 34 (46.6%) were on antibiotic therapy when they were included in the study (Table 1).

**Table 1 -:** Comparative analysis of the characteristics of the patients in the groups studied.a

Variable	BAL	EA	p
	(n = 36)	(n = 37)	
Male/female, n/n	15/21	11/26	0.287*
Age, years	67.1 ± 13.9	64.5 ± 14.8	0.438***
Duration of MV before VAP, h^b^	155.5 (111.0-270.0)	192.0 (108.0-384.0)	0.429**
Comorbidities, n	7	6	0.719*
Previous use of antibiotics^c^	16 (44.0)	18 (48.6)	0.719*
Duration of previous antibiotic therapy, days^b,d^	6.0 (1.0-10.0)	6.0 (1.7-12.3)	0.721**
Body temperature, °C^b^	38.5 (38.0-38.7)	38.4 (38.0-38.5)	0.481**
Mean arterial pressure^b^	80.0 (67.5-90.7)	79.0 (71.0-96.5)	0.440**
Leukocyte count, cells/mm^3b^	13,250 (9,525-16,200)	12,700 (9,950-16,300)	0.851**
PaO_2_/FiO_2_^b^	197.5 (134.3-267.5)	205.0 (154.0-274.0)	0.651**
APACHE II	17.5 ± 5.7	15.3 ± 6.9	0.146***
APACHE II predicted mortality^b^	19.5 (10.1-29.1)	13.4 (7.7-32.2)	0.477**
Blood culture			0.758*
Not performed^c^	2 (5.6)	1 (2.7)	
Negative^c^	22 (61.1)	25 (67.6)	
Positive^c^	12 (33.3)	11 (29.7)	
Negative/positive, n/n	22/12	25/11	0.867*

EA: endotracheal aspiration; MV: mechanical ventilation; VAP: ventilator-associated pneumonia; and APACHE II: Acute Physiology and Chronic Health Evaluation II. aValues expressed as mean ± SD, except where otherwise indicated. bValues expressed as median (interquartile range). cValues expressed as n (%). dn = 16 and n = 18 in the BAL and EA groups, respectively.

* Chi-square test;

** Mann-Whitney test; and

*** Student's t-test.

The mean volume of BALF collected was 34.33 ± 14.08 mL, and the mean results were 2,859.78 ± 7,635.50 cells/mL and 74.14 ± 31.3% of neutrophils. In 24 (66.7%) of the BALF samples, the mean proportion of cells containing ICOs was 3.00 ± 3.37% cells. Of the 36 BALF samples, 35 (97.2%) yielded positive quantitative cultures. On the basis of these values, the presence of cells containing ICO had a sensitivity of 0.69, a specificity of 1.0, a positive predictive value (PPV) of 1.0, and a negative predictive value (NPV) of 0.08 in relation to the presence of positive quantitative BALF cultures. The most commonly isolated bacteria in the BALF cultures were* P. aeruginosa*, in 9 samples (25.0%),* K. pneumoniae*, in 8 (22.2%); and MRSA, in 2 (5.6%). *C. albicans* was present in 11 cultures (30.6%).

Cells containing ICOs were found in 21 (56.8%) of the endotracheal aspirate samples, with a mean value of 4.3 ± 10.3% cells (range, 0-60%). Of the 37 QEACs, 36 (97.3%) were positive. Accordingly, the presence of ICOs showed a sensitivity of 0.59, a specificity of 0.0, a PPV of 0.98, and an NPV of 0.0 for the presence of VAP (positive QEAC being used as reference). The most commonly isolated bacteria in the endotracheal aspirate cultures were* K. pneumoniae*, in 12 samples (32.4%);* P. aeruginosa*, in 8 (21.6%), MRSA, in 5 (13.5%); *S. pneumoniae*, in 2 (5.4%); and others, in 27 (73.0%). *C. albicans* was identified in 14 samples (37.8%). 

Regarding the primary outcome, the mortality rate at 28 days was 25.0% and 37.8% in the BAL and EA groups, respectively (p = 0.353; Table 2). The overall mortality and the mortality at 14 days were also similar (Table 2).

**Table 2 -:** Mortality rates in the groups studied.

Mortality	BAL	EA	p*
	(n = 36)	(n = 37)	
At 14 days	8 (22.2)	11 (29.7)	0.643
At 28 days	9 (25.0)	14 (37.8)	0.353
Overall^b^	19 (52.8)	23 (62.2)	0.417

EA: endotracheal aspiration.

a Values expressed as n (%).

b It includes patients that died beyond 28 days after inclusion.

* Chi-square test.

Regarding the secondary outcomes, the median duration of antibiotic therapy was 14 days in both groups. In almost 78% of the patients in the BAL group and in 83% of those in the EA group, the initial antibiotic therapy was deemed appropriate (p = 0.551), and mortality was similar in this subset of patients (35.7% vs. 43.3%, respectively; p = 0.553). When we compared the patients (of both groups) who received appropriate therapy with those who did not, mortality was also similar. The initial antibiotic regimens were modified in 87.5% and 100.0% of the patients with inappropriate antibiotic therapy in the BAL and EA groups (p = 1.0), respectively, mostly due to the microbiological results; escalation of therapy was necessary in 34.8% and in 38.1% of the patients in the BAL and EA groups, respectively, and was similar (p = 0.82). Median duration of antibiotic therapy, length of ICU stay, proportion of patients with appropriate antibiotic therapy, and mortality rates in the appropriate and inappropriate subgroup of patients, as well as the proportion and causes of modifications in the therapy, were also similar (Tables 3 and 4). Adverse events were all transient and expeditiously managed in accordance with specific recommendations. In the BAL group, no adverse events were found in 27 patients (75.0%); desaturation < 90% occurred in 2 (5.6%); transient hypotension occurred in 1 (2.8%); and sinus tachycardia > 120 bpm occurred in 7 (19.4%). In the EA group, there were no serious adverse events in 32 patients (86.5%), and all complications were transient and of no clinical significance: there were desaturation < 90% in 3 patients (8.1%), sinus tachycardia in 1 (2.7%), and bradycardia in 1 (2.7%).

**Table 3 -:** Comparison of secondary outcomes in the groups studied.a

Variable	BAL	EA	p
	(n = 36)	(n = 37)	
Antibiotic therapy, days	14.0 (13.3-17.0)	14.0 (10.5-20.0)	0.708**
Length of ICU stay, days^b^			
≤ 14	8 (22.2)	8 (21.6)	0.797*
15-27	12 (33.3)	15 (40.5)	
≥ 28	16 (44.5)	14 (37.9)	
Length of mechanical ventilation, days	16.0 (9.7-32.5)	19.5 (12.0-33.2)	0.597**
Appropriate antibiotic therapy^b^	28 (77.8)	30 (83.3)	0.551*
Mortality^b,d^			
Appropriate therapy	10 (35.7)	13 (43.3)	0.553*
Inappropriate therapy	5 (62.5)	3 (50.0)	1.000***
Antibiotic therapy changes after culture^b,d^			
Appropriate therapy	16 (57.1)	16 (53.3)	0.771*
Inappropriate therapy	7 (87.5)	6 (100.0)	1.000***
Reasons for antibiotic change (appropriate group)^c,e^			
BALF/endotracheal aspirate results	15	13	0.498***
Another site of infection	1	2	
Clinical deterioration	0	2	
Reasons for antibiotic change (inappropriate group)^c,f^			
BALF/endotracheal aspirate results	6	4	0.706***
Another site of infection	1	1	
Clinical deterioration	0	1	

EA: endotracheal aspiration; and BALF: bronchoalveolar lavage fluid.

a Values expressed as median (interquartile range), except where otherwise indicated.

b Values expressed as n (%).

c Values expressed as n.

d In relation to the appropriateness of the therapy: appropriate treatment: n = 28 and n = 30 in the BAL and EA groups, respectively; and inappropriate treatment: n = 8 and n = 6 in the BAL and EA groups, respectively.

e n =16 and n = 17 in the BAL and EA groups, respectively.

f n = 7 and n = 6 in the BAL and EA groups, respectively.

* Chi-square test;

** Mann-Whitney test; and

*** Fisher's exact test.

**Table 4 -:** Comparison of outcomes in relation to the appropriateness of the antibiotic therapy in the groups studied.a

Variable	Group					
	BAL			EA		
	AAT	IAT	p	AAT	IAT	p
Duration of antibiotic therapy, days	14 (14.0-17.0)	14 (3.5-16.3)	0.348*	15 (11.7-20.0)	12 (7.0-21.0)	0.622*
Length of ICU stay, days^b^						
≤ 14	7 (25.0)	1 (12.5)	0.775**	6 (20.0)	1 (16.7)	0.851**
15-27	9 (32.1)	3 (37.5)		13 (43.3)	2 (33.3)	
≥ 28	12 (42.9)	4 (50.0)		11 (36.7)	3 (50.0)	
Antibiotic therapy changes after culture^b^	16 (57.1)	7 (53.3)	0.213**	16 (53.3)	6 (100.0)	0.062**

EA: endotracheal aspiration; AAT: appropriate antibiotic therapy; and IAT: inappropriate antibiotic therapy. aValues expressed as n (%) or as median (interquartile range).

* Mann-Whitney test; and

** Fisher's exact test.

Similarly, the proportions of responsive patients and of the occurrence of sepsis, severe sepsis, septic shock, secondary VAP, and noninfectious complications, as well as the need for other diagnostic procedures (data not shown) were evenly distributed in both groups (Table 5).

**Table 5 -:** Secondary clinical outcomes in the groups studied. a

Variable	BAL	EA	p
Responders to the modified antibiotic therapy^b^	23 (92.0)	19 (79.2)	0.247**
Secondary complications			
SIRS^c^	3 (75.0)	2 (66.7)	1.000**
Sepsis^d^	20 (95.2)	20 (95.2)	1.000**
Severe sepsis^e^	5 (20.0)	2 (10.5)	0.680**
Septic shock^f^	10 (28.6)	15 (45.5)	0.149*
Secondary VAP^g^	0 (0.0)	2 (5.6)	0.151*
Organ failure^g^	5 (13.9)	9 (25.0)	0.234*
Non-infectious complications^g^	4 (11.1)	4 (11.1)	0.645**
Second EA^h^	0 (0.0)	4 (36.4)	0.085**
Second BAL^h^	0 (0.0)	0 (0.0)	-
Blood culture^h^	2 (25.0)	2 (18.2)	1.000**

EA: endotracheal aspiration; SIRS: systemic inflammatory response syndrome; and VAP: ventilator-associated pneumonia.

a Values expressed as n (%).

b n = 25 and n = 24 in the BAL and EA groups, respectively.

c n = 4 and n = 3 in the BAL and EA groups, respectively.

d n = 21 in both groups.

e n = 25 and n = 19 in the BAL and EA groups, respectively.

f n = 35 and n = 33 in the BAL and EA groups, respectively.

g n = 36 in both groups.

h n = 8 and n = 11 in the BAL and EA groups, respectively.

* Chi-square test; and

** Fisher's exact test.

## Discussion

The major findings of the present study were as follows: the mortality rates at 28 days after the inclusion of the patients in the study were similar, independently of the diagnostic strategy used; most patients in both groups were prescribed appropriate antibiotic therapy (78% and 83% of the patients in the BAL and EA groups, respectively), and the mortality rates were similar in these subsets of patients; other secondary outcomes were all proportionally similar, such as length of hospital and ICU stay, duration of antibiotic therapy, modification in antibiotic therapy after culture results, and use of additional diagnostic techniques, as well as the occurrence of sepsis, septic shock, secondary VAP, and organ failure.

The low accuracy of the clinical diagnosis of VAP has prompted the use of fiberoptic bronchoscopy and quantitative cultures of respiratory samples in order to discriminate colonization from true infection in mechanically ventilated ICU patients, which has been supported by studies showing acceptable concordance among BALF, PSB, and postmortem pulmonary biopsy cultures.^(^ 8 ^,^ 14 ^-^ 16 ^)^

In one study, EA was considered a useful diagnostic method because of its similarity with BAL in terms of microbial diagnosis and clinical outcomes, especially when endotracheal aspirate samples are quantitatively cultured.^(^ 17 ^)^ However, robust evidence favoring one of these methods in the management of VAP patients is still lacking. ^(^ 9 ^-^ 11 ^,^ 18 ^-^ 20 ^)^ The adoption of invasive or noninvasive strategies has not resulted in clear differences in terms of outcomes when quantitative cultures are performed.^(9-11,21,22) ^One group of authors reported that patients with suspected VAP who had been managed by means of BAL showed lower 14-day mortality rate, earlier resolution, earlier attenuation of organ failure, and less antibiotic use than did controls managed by means of QEAC; however, those differences could have been influenced by the use of qualitative cultures in the endotracheal aspirate arm of the study, which prevented any adjustments to the therapy.^(^ 9 ^)^ In two studies,^(^ 10 ^,^ 11 ^)^ similar clinical outcomes were found concerning the use of quantitative BAL, PSB, and EA techniques, except for a higher frequency of therapeutic modifications in the quantitative BAL and PSB groups. No significant differences were found in terms of mortality rates. One group of investigators also found that quantitative BALF cultures and nonquantitative endotracheal aspirate cultures were associated with similar clinical outcomes and use of antibiotics; however, some drawbacks of their research were the exclusion of patients with MRSA or *Pseudomonas* sp., the nonquantitative culture methodology used in the endotracheal aspirate samples, and the use of broad-spectrum antibiotics in both groups, which could have limited the strength of their results. ^(^ 23 ^)^ In addition, a meta-analysis demonstrated no evidence that the use of invasive strategies could result in reduced mortality, shorter length of ICU stay, shorter duration of MV, or higher rates of antibiotic regimen changes when compared with the use of noninvasive strategies.^(^ 24 ^)^

In spite of the limitations of the use of mortality rates as the endpoint in studies involving ICU patients,^(^ 2 ^,^ 25 ^,^ 26 ^)^ the present study reinforces the concept that the mortality rate involves multifactorial variables, and it appears to be independent of diagnostic techniques used for collection of respiratory samples in VAP patients as long as a comprehensive protocol is appropriately followed.^(^ 7 ^,^ 10 ^,^ 17 ^)^

One important cofactor is the previous use of antibiotics, a usual context at the time of suspicion of VAP. It has been accepted that this might impair the yield of cultures and is associated with higher VAP mortality and selection of potentially resistant bacteria.^(^ 13 ^,^ 15 ^,^ 27 ^-^ 30 ^)^ In our series, almost half of our patients were on antibiotic therapy at the time of enrollment (44.0% and 48.6% in the BAL and EA groups, respectively). Similarly, it was associated with the isolation of resistant microorganisms. However, a negative impact of this variable on the yield of cultures was not found, which is in agreement with the available literature. This finding could be explained by the overall median of 6 days of previous antibiotic use, which is longer than the limit of 3 days reported in the literature as the point of no interference with culture yield.^(^ 28 ^,^ 31 ^)^

Although adjustments in the antibiotic regimens were similarly carried out in both groups, they had no impact on mortality rates, length of ICU stay, length of hospital stay, duration of antibiotic therapy, duration of MV, or rate of complications, regardless of the procedure adopted.

Our findings refer to a single ICU, which might not reflect other critical health care contexts. Because VAP still lacks a gold standard method of diagnosis, the therapeutic modifications were not exclusively based on the microbiological results. In addition, as it has been reported on in other studies,^(^ 8 ^,^ 15 ^)^ we might have included false positive and false negative cases. Nevertheless, the microbial identification might locally contribute to the optimal management of VAP patients by recognizing the local flora and further enhancing the rates of appropriate therapy.

In conclusion, we found no differences in the 28-day mortality rates or other clinical outcomes that could be linked to the diagnostic methods used in this study for the confirmation of VAP. Therefore, QEAC can be considered a practicable tool in the microbiological diagnosis of VAP. The prior use of antibiotics, in similar contexts, might not interfere with the yield of respiratory cultures.
